# Genome-wide identification and gene expression analysis of *SOS* family genes in tuber mustard (*Brassica juncea* var. *tumida*)

**DOI:** 10.1371/journal.pone.0224672

**Published:** 2019-11-11

**Authors:** Chunhong Cheng, Yuanmei Zhong, Qing Wang, Zhaoming Cai, Diandong Wang, Changman Li

**Affiliations:** School of Advanced Agriculture and Bioengineering, Yangtze Normal University, Chongqing, P.R. China; ICAR-National Research Centre on Plant Biotechnology, INDIA

## Abstract

The Salt Overly Sensitive (SOS) pathway in *Arabidopsis thaliana* plays important roles in maintaining appropriate ion homeostasis in the cytoplasm and regulating plant tolerance to salinity. However, little is known about the details regarding SOS family genes in the tuber mustard crop (*Brassica juncea* var. *tumida*). Here, 12 *BjSOS* family genes were identified in the *B*. *juncea* var. *tumida* genome including two homologous genes of *SOS1*, one and three homologs of *SOS2* and *SOS3*, two homologs of *SOS4*, two homologs of *SOS5* and two homologs of *SOS6*, respectively. The results of conserved motif analysis showed that these SOS homologs contained similar protein structures. By analyzing the *cis*-elements in the promoters of those *BjSOS* genes, several hormone- and stress-related *cis*-elements were found. The results of gene expression analysis showed that the homologous genes were induced by abiotic stress and pathogen. These findings indicate that *BjSOS* genes play crucial roles in the plant response to biotic and abiotic stresses. This study provides valuable information for further investigations of *BjSOS* genes in tuber mustard.

## Introduction

Tuber mustard, *Brassica juncea* var. *tumida* (AABB, 2n = 36), which belongs to *Brassicaceae*, is an allotetraploid species that was produced from a natural cross between *B*. *rapa* (AA, 2n = 20) and *B*. *nigra* (BB, 2n = 16), followed by chromosome doubling [1]. It is an important vegetable in China and some Southeast Asian countries, as it serves as the raw material for Fuling mustard and is also famous for its special flavor and nutritional value. However, during growth and development, tuber mustard frequently suffers from abiotic and biotic stresses, such as salinity and pathogens, resulting in the inhibition of plant growth and huge economic loss. Therefore, determining the mechanisms underlying its resistance to salt stress will be helpful for improving the production of this vegetable.

The Salt Overly Sensitive (SOS) signaling pathway plays important roles in the plant response to salt stress and has three components: SOS1, SOS2 and SOS3. *SOS1* encodes a Na^+^/H^+^ anti-transport protein in the cell membrane, which transports excess Na^+^ from the cytoplasm to the extracellular region [2]; *SOS2* encodes a serine/threonine protein kinase; and *SOS3* encodes a Ca^2+^ binding protein [3, 4]. Under salt stress, the concentration of Ca^2+^ in the cytoplasm immediately increases and is perceived by SOS3. SOS3 further activates SOS2 protein kinase by combining with Ca^2+^. SOS2 can interact with SOS3 and forms the SOS2-SOS3 complex to regulate the expression of SOS1 by phosphorylation. The transport activity of SOS1 is activated by SOS2-SOS3, and excess Na^+^ is discharged to alleviate the toxic effects of Na^+^ on cells [4–6]. Besides that, AtSOS4, AtSOS5 and AtSOS6 were also identified using a root-bending assay for the regulation of ion homeostasis and cell expansion under salt stress [7–10]. *AtSOS4* encodes a pyridoxal kinase which is involved in the biosynthesis of vitamin B6 and regulates Na^+^ and K^+^ homeostasis [7, 8]. *AtSOS5* encodes a putative cell surface adhesion protein and is required for normal cell expansion [9]. The root tips of *sos5* mutant swell and root growth is arrested [9]. *AtSOS6* encodes a cellulose synthase-like protein, AtCSLD5 [10]. The *sos6-1* mutant shows hypersensitive to salt stress and osmotic stress, and accumulates high level of reactive oxygen species (ROS) [10].

The *Arabidopsis* mutants *sos1*, *sos2* and *sos3* are hypersensitive to Na^+^ and Li^+^, indicating that SOS proteins are involved in the regulation of plant tolerance to salinity [11]. Transgenic *Arabidopsis* seedlings overexpressing *SOS1* show enhanced tolerance to salt stress and less Na^+^ content compared to wild-type plants under NaCl treatment [12]. The lateral root development of the *sos3-1* mutant shows increased sensitivity even at low salt concentration, confirming that the SOS signaling pathway also modulates organ development in response to salt stress [13]. Overexpression of *B*. *juncea SOS3* (*BjSOS3*) in the *Arabidopsis* mutant *sos3* complements the *sos3* mutant phenotype and transgenic plants exhibit enhanced tolerance to salinity, indicating that BjSOS3 has conserved function with AtSOS3 in regulating plant resistance to salt stress [13, 14]. However, the role of the SOS gene family in tuber mustard remains mostly unknown. Therefore, we identified the *SOSs* genes and elucidated their putative role for better understanding of SOS signaling pathway in tuber mustard (*Brassica juncea* var. *tumida*).

In this study, we identified 12 *SOS* family genes in the *B*. *juncea* var. *tumida* genome. Based on the analysis of phylogenic relationship, gene structures, protein motifs, and promoter *cis*-elements, similar gene characteristics were found between *BjSOS* and *AtSOS*. In addition, we analyzed the transcript levels of *BjSOS* family genes under biotic and abiotic stresses, including NaCl, ABA, low temperature, and the pathogen *Plasmodiophora Brassicae*. The results showed that *BjSOS* genes were induced by abiotic stresses and pathogen in tuber mustard. The findings indicate that SOS family genes play crucial roles in the plant response to biotic and abiotic stresses. The findings not only are helpful for further understanding of the SOS signaling pathway but also provide clues about the defense responses of tuber mustard against different stresses.

## Materials and methods

### Materials and growth conditions

The tuber mustard cultivar Yong’an was used in this study. The seeds were surface sterilized and plated on MS medium (Sigma-Aldrich, St. Louis, MO, USA) with 1% sucrose and 8 g/L agar (Sigma-Aldrich, St. Louis, MO, USA) and then cultivated in a growth room at 22°C and 6000 lx under long-day conditions (16 h light/8 h dark). To analyze the gene expression patterns of *BjSOS* genes under abiotic stresses, 1-week-old seedlings grown on MS medium were treated with 50 μM ABA, 200 mM NaCl and low temperature (4°C) for the indicated time points. To analyze the gene expression patterns of *BjSOS* genes under biotic stress, 2-week-old seedlings were irrigated with *P*. *brassicae* suspension liquid (OD_600_ = 0.07) for 0, 0.25, 0.5, 1, 3, 5, 7, and 9 days.

### Bioinformatics analysis

The gene sequences of *AtSOS1*, *AtSOS2*, *AtSOS3*, *AtSOS4*, *AtSOS5* and *AtSOS6* and their homologous genes in tuber mustard were searched in the Phytozome (https://phytozome.jgi.doe.gov/pz/portal.html), TAIR (http://www.arabidopsis.org/) and Brassica databases (http://brassicadb.org/brad/). The protein sequences were aligned by ClustalX 1.83 [15], and a phylogenic tree was constructed using the neighbor-joining method with bootstrap values of 1000 by MEGA5 [16]. Gene structure analysis was performed using online software (http://gsds.cbi.pku.edu.cn/). The regions located 2 kb upstream of the *BjSOS* coding sequences were used as the promoter sequences, and the promoter *cis*-element analysis was performed using PlantCARE (http://bioinformatics.psb.ugent.be/webtools/plantcare/html/) and PLACE (https://sogo.dna.affrc.go.jp/cgi-bin/sogo.cgi?lang=en&pj=640&action=page&page=newplace) online software. Protein domain analysis was done using SMART (http://smart.embl-heidelberg.de/) and ExPASy (http://prosite.expasy.org/prosite.html) online analysis tools.

### Gene expression analysis

Total RNA was extracted from tuber mustard seedlings that had been subjected to NaCl, ABA, low temperature, and pathogen treatment using TRIzol reagent (Invitrogen, Carlsbad, CA, USA; Catalog No. 15596026). The RNA samples were used for cDNA synthesis using the cDNA synthesis Supermix with gDNA remover kit (Transgen Biotech, China; Catalog No. AT301) following the manufacturer’s instructions. qRT-PCR was carried out using SYBR Green qPCR Supermix (Invitrogen, Carlsbad, CA, USA; Catalog No. 4309155). The transcript abundance was calculated by the comparative *C*_*T*_ (cycle threshold) method, and *BjActin3* was used as the internal control. The qRT-PCR experiments were carried out three times with three replicates each. The primers used in this study were listed in S1 Table.

### Statistical analysis

All data were analyzed using SigmaPlot 10.0 (Systat Software, Inc., Chicago, IL) and SPSS 16.0 software. The averages and standard deviations of all results were calculated, and for multiple groups of samples, the one-way ANOVA followed by the Dunnett test was used. The statically significant treatments were marked with ‘***’ (P<0.001), ‘**’ (P<0.01) and ‘*’ (P<0.05).

## Results

### Genome-wide identification and characterization of SOS homologs in *B*. *juncea* var. *tumida*

**Fig 1:**
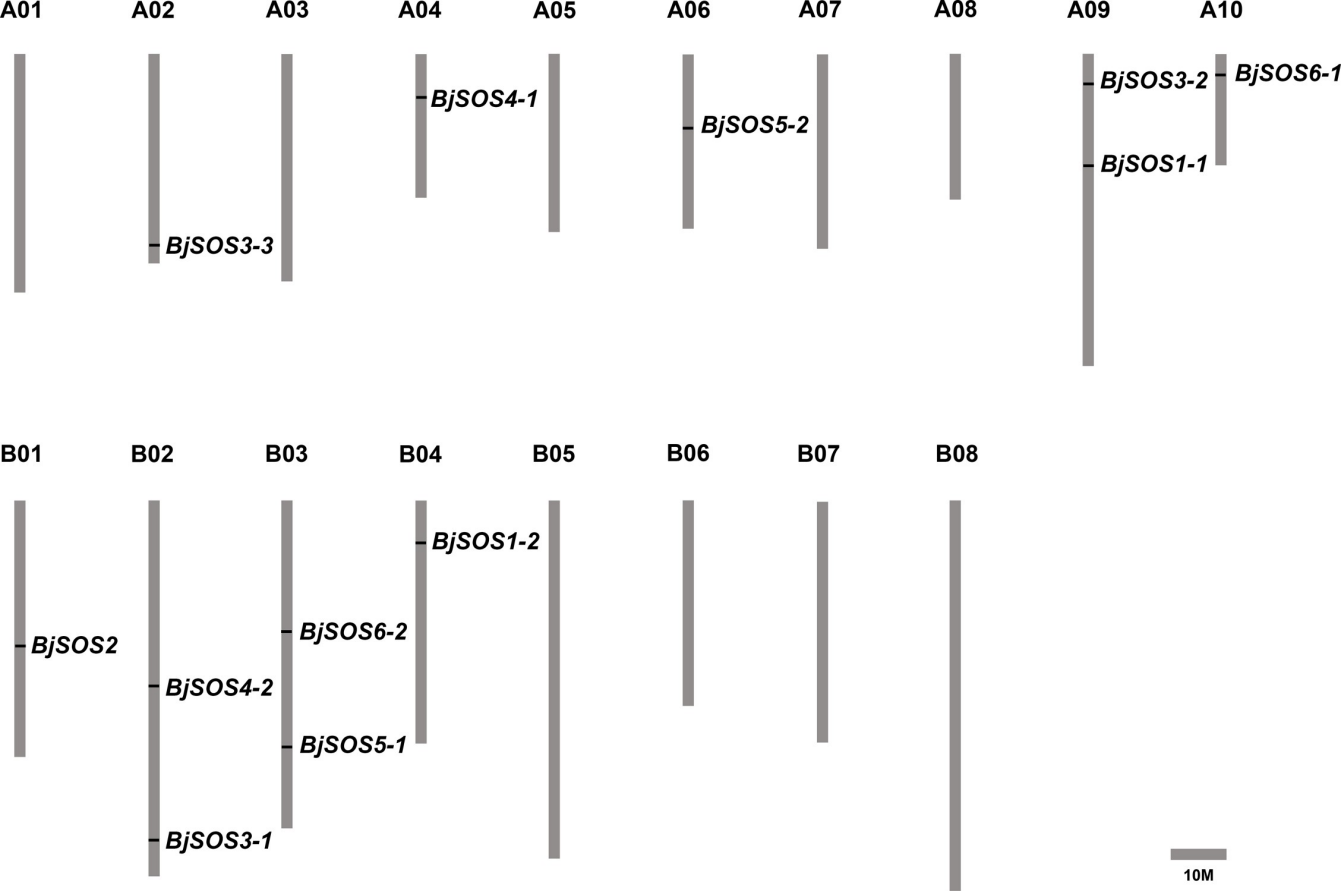
The distribution of *BjSOS* in *Brassica juncea* var. *tumida* chromosomes. Twelve identified SOS homologs genes were mapped to the 9 of 18 chromosomes. The chromosome name is at the top of each bar. The scale of the chromosome is in millions of bases (Mb).

Twelve genes as homologs of SOS genes were identified in *B*. *juncea* var. *tumida* genome through BLASTP in *Brassica* database using six AtSOS protein sequences as references (Table 1). The gene lengths ranged from 1191 bp to 5965 bp with 1–23 exons in each sequence. The protein lengths of these twelve SOS homologs ranged from 180 (*BjSOS3-3*) to 1192 (*BjSOS6-2*) amino acid (aa) residues. The relative molecular weights of those proteins varied from 20.59 kD (BjSOS3-3) to 133.61 kD (BjSOS6-2), and the isoelectric point (PI) ranged from 4.90 to 9.25 (Table 1). The twelve *BjSOS* genes were distributed in 9 of the 18 chromosomes of *B*. *juncea* var. *tumida*. Each of the chromosomes A02, A04, A06, A10, B01, and B04 contained one gene, and each of the chromosomes A09, B02 and B03 contained two genes (Fig 1).

**Table 1:** The SOS family members in *Brassica juncea* var. *Tumida*.

Group	Gene name	Locus	Sequence ID	Exon	Genomics (bp)	CDS (bp)	Protein (aa)	pI	MW (kD)
SOS1	*BjSOS1-1*	A09	*BjuA002024*	23	5789	3030	1009	6.82	111.85
	*BjSOS1-2*	B04	*BjuB027801*	23	5965	3321	1106	6.21	122.13
SOS2	*BjSOS2*	B01	*BjuB024452*	12	2716	1221	406	9.25	46.16
SOS3	*BjSOS3-1*	B02	*BjuB038085*	8	1418	660	219	5.04	25.35
	*BjSOS3-2*	A09	*BjuA046788*	8	1492	657	218	4.90	25.13
	*BjSOS3-3*	A02	*BjuA033133*	7	1191	543	180	5.08	20.59
SOS4	*BjSOS4-1*	A04	*BjuA015607*	12	2615	930	309	5.34	34.16
	*BjSOS4-2*	B02	*BjuB048173*	12	2627	930	309	5.11	34.06
SOS5	*BjSOS5-1*	B03	*BjuB019581*	1	1269	1272	423	5.68	44.16
	*BjSOS5-2*	A06	*BjuA022634*	1	1260	1263	420	5.52	44.22
SOS6	*BjSOS6-1*	A10	*BjuA037683*	3	3697	3534	1177	8.21	132.02
	*BjSOS6-2*	B03	*BjuB043857*	3	3784	3579	1192	7.85	133.61

pI: Isoelectric point; MW: molecular weight.

### Phylogenic analysis and gene structures of SOS family genes

**Fig 2:**
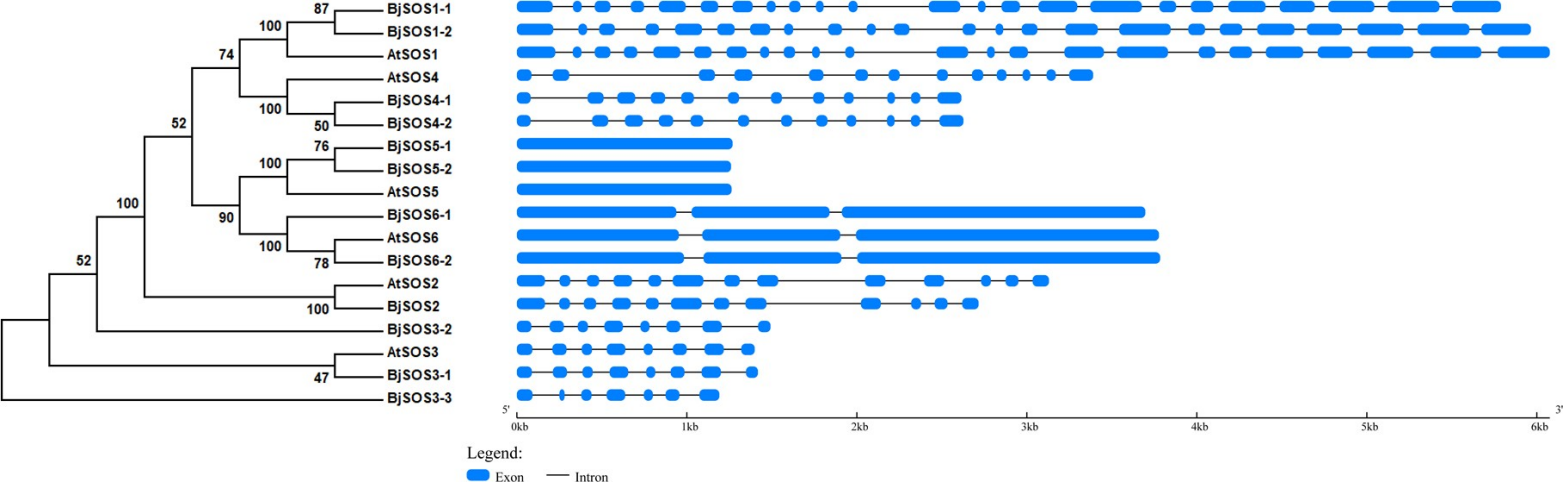
The phylogenic tree and gene structures of SOS family genes. The phylogenic tree was built using the neighbor-joining (NJ) method and the exon-intron structure of SOS homologs was drawn according to their phylogenic relationships. The blue boxes and gray lines denoted exons and introns, respectively.

To analyze the evolutionary relationships between BjSOSs and AtSOSs, a phylogenetic tree was constructed using MEGA5 software with the neighbor-joining method. According to the phylogenic tree, twelve *BjSOS* genes with six *AtSOS* genes were identified and clustered into six clades. The first clade was *AtSOS1* and two homologs *BjSOS1-1* and *BjSOS1-2*; the second clade was *AtSOS4* and two homologs *BjSOS4-1* and *BjSOS4-2*; the third clade was *AtSOS5* and its two homologs *BjSOS5-1* and *BjSOS5-2*; the fourth clade was *AtSOS6* and its two homologs *BjSOS6-1* and *BjSOS6-2*; the fifth clade was *AtSOS2* and its homolog *BjSOS2*; and the last clade was *AtSOS3* and its homologs *BjSOS3-1*, *BjSOS3-2* and *BjSOS3-3* (Fig 2). *SOS* family genes in the same subfamilies may have similar functions. To understand their gene structures, we analyzed the gene exon-introns using the GSDS2.0 online server. According to the results, *AtSOS1*, *BjSOS1-1* and *BjSOS1-2* all had 23 exons and 22 introns; *AtSOS2* and *BjSOS2* contained 13 and 12 exons, respectively; *AtSOS3*, *BjSOS3-1* and *BjSOS3-2* all contained eight exons with the exception of *BjSOS3-3* (7 exons); *AtSOS4*, *BjSOS4-1* and *BjSOS4-2* contained 13 and 12 exons, respectively; *AtSOS5*, *BjSOS5-1* and *BjSOS5-2* all contained one exon; *AtSOS6*, *BjSOS6-1* and *BjSOS6-2* all contained 3 exons (Fig 2). These results indicated that the homologs clustered into the same subfamily had similar gene structures and might have conserved functions.

### Protein sequence alignment and conserved motif analysis of SOS homologs

**Fig 3:**
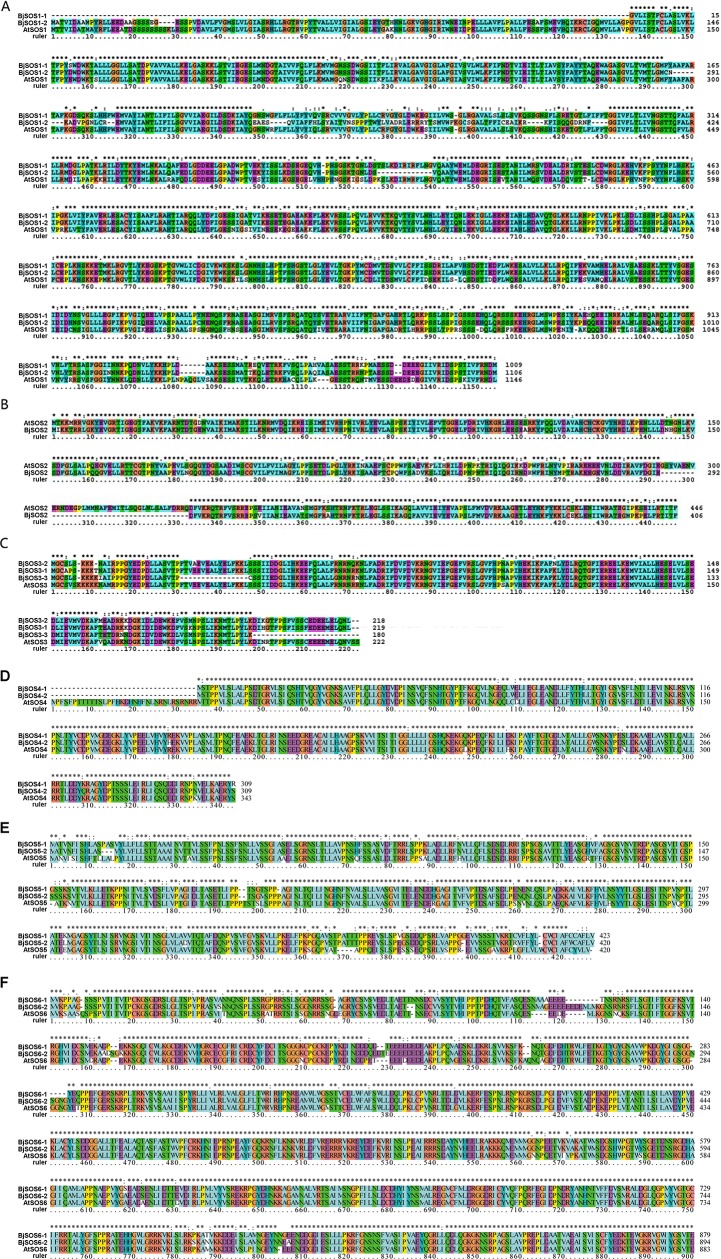
The protein sequence alignment of SOS homologs. The protein sequences were aligned by Clustalx. A. The protein sequence alignment of AtSOS1 and its homologs. B. The protein sequence alignment of AtSOS2 and its homologs. C. The protein sequence alignment of AtSOS3 and its homologs. D. The protein sequence alignment of AtSOS4 and its homologs. E. The protein sequence alignment of AtSOS5 and its homologs. F. The protein sequence alignment of AtSOS6 and its homologs.

The SOS homologous protein sequences were aligned by Clustalx and the results showed that homologs clustered into the same subfamily had conserved protein sequences (Fig 3). BjSOS1-1 and BjSOS1-2 shared 73.11% and 75.74% sequence identity, respectively, with AtSOS1; AtSOS2 and BjSOS2 shared 81.39% sequence identity; AtSOS3 and its homologs BjSOS3-1, BjSOS3-2, and BjSOS3-3 shared 89.19%, 88.29%, and 72.07% sequence identity, respectively; BjSOS4-1 and BjSOS4-2 shared 91% and 90% sequence identity with AtSOS4, respectively; BjSOS5-1 and BjSOS5-2 shared 74% and 73% sequence identity with AtSOS5, respectively; and BjSOS6-1 and BjSOS6-2 shared 88% and 87% sequence identity with AtSOS6, respectively (Fig 3).

**Fig 4:**
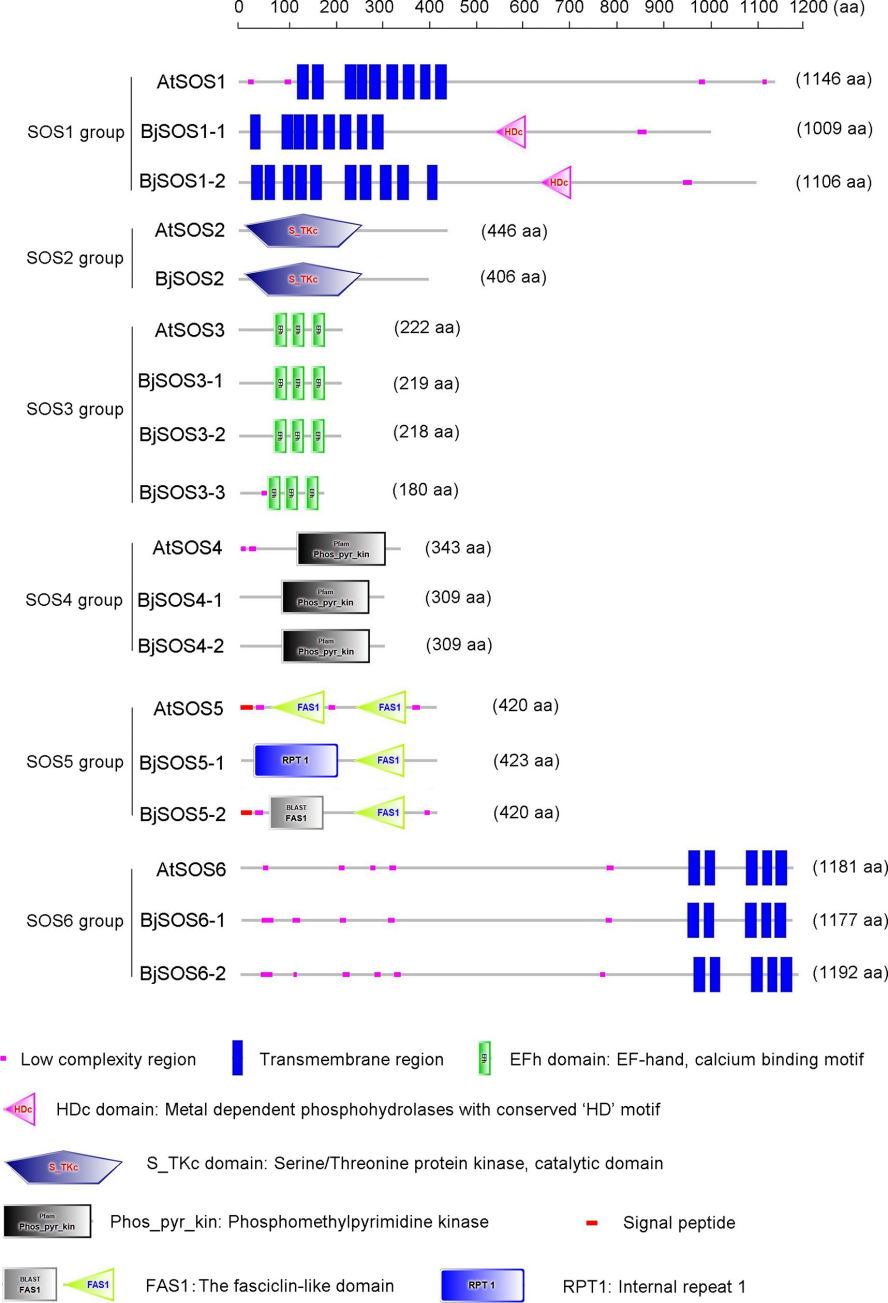
The conserved motifs of the SOS homologs proteins. These motifs were identified using the online software of SMART, and colored boxes indicated conserved motifs and gray lines represent non-conserved sequences.

Protein conserved motif analysis was conducted using the SMART and ExPASy online analysis tools. The results showed that AtSOS1 and its homologs BjSOS1-1 and BjSOS1-2 all contained 8–10 transmembrane regions; both BjSOS1-1 and BjSOS1-2 contained 1 HDc domain; both AtSOS2 and BjSOS2 contained the S_TKc domain, which was a serine/threonine protein kinase catalytic domain; AtSOS3 and its homologs BjSOS3-1, BjSOS3-2, and BjSOS3-3 all contained the EFh domain, which was calcium Ca^2+^- binding motif; AtSOS4, BjSOS4-1 and BjSOS4-2 all contained Phos_pyr_kin domain, which was a phosphomethylpyrimidine kinase domain; AtSOS5, BjSOS5-1 and BjSOS5-2 all contained FAS1 domain, which was the fasciclin-like domain; AtSOS6, BjSOS6-1 and BjSOS6-2 all contained 5 transmembrane regions (Fig 4). The results indicated that the BjSOS homologs might have conserved functions in response to abiotic stress.

### Promoter cis-acting regulatory elements prediction of SOS homologs

**Fig 5:**
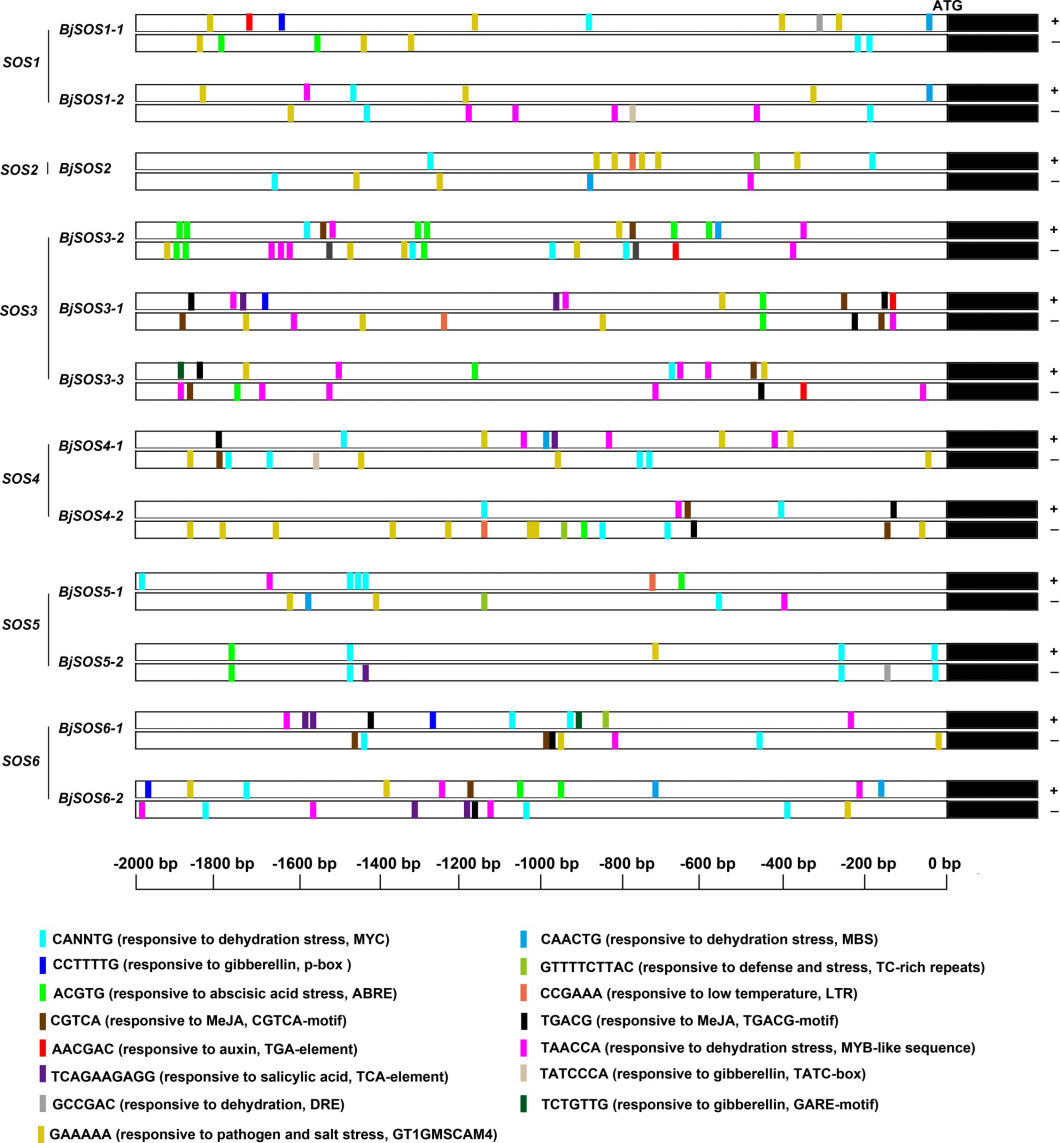
The promoter *cis*-elements analysis of SOS homologs genes. The 2 kb DNA fragments upstream of the ATG staring code of SOS homologs genes were analyzed using online analysis software PlantCARE (http://bioinformatics.psb.ugent.be/webtools/plantcare/html/) and PLACE (https://sogo.dna.affrc.go.jp/cgi-bin/sogo.cgi?lang=en&pj=640&action=page&page=newplace).

To further understand the potential roles of SOS homologs and how their gene expression is regulated, we chose the 2000 bp DNA fragment upstream of the ATG start codon as the promoter sequences and performed promoter *cis*-element analysis using PlantCARE and PLACE online software. According to the results, the promoters of all *BjSOS* genes, except *BjSOS2*, contained at least one hormone-related elements such as the ABRE (ACGTG, responsive to abscisic acid stress) [17], p-box (CCTTTTG, responsive to gibberellin) [18], and CGTCA motif (CGTCA, responsive to MeJA) [19] (Fig 5 and S2 Table). In addition, the promoters of the SOS homologs contained at least eight stressed-related elements such as the DRE (GCCGAC, responsive to dehydration) [20], MBS (CAACTG, responsive to dehydration stress) [21], TC-rich repeats (GTTTTCTTAC, responsive to defense and stress) [22], LTR (CCGAAA, responsive to low temperature) [23], and GT1GMSCAM4 motif (GAAAAA, responsive to pathogen and salt stress) [24] (Fig 5 and S2 Table). Together, the promoters of SOS homologs contained diverse *cis*-elements responsive to ABA, auxin, GA, SA, and abiotic stresses, indicating that the genes expression of SOS homologous were regulated by hormone and abiotic stresses, and they might play a role in regulating tuber mustard response to hormone and stresses.

### Tissue specific expression pattern analysis of SOS homologs genes

**Fig 6:**
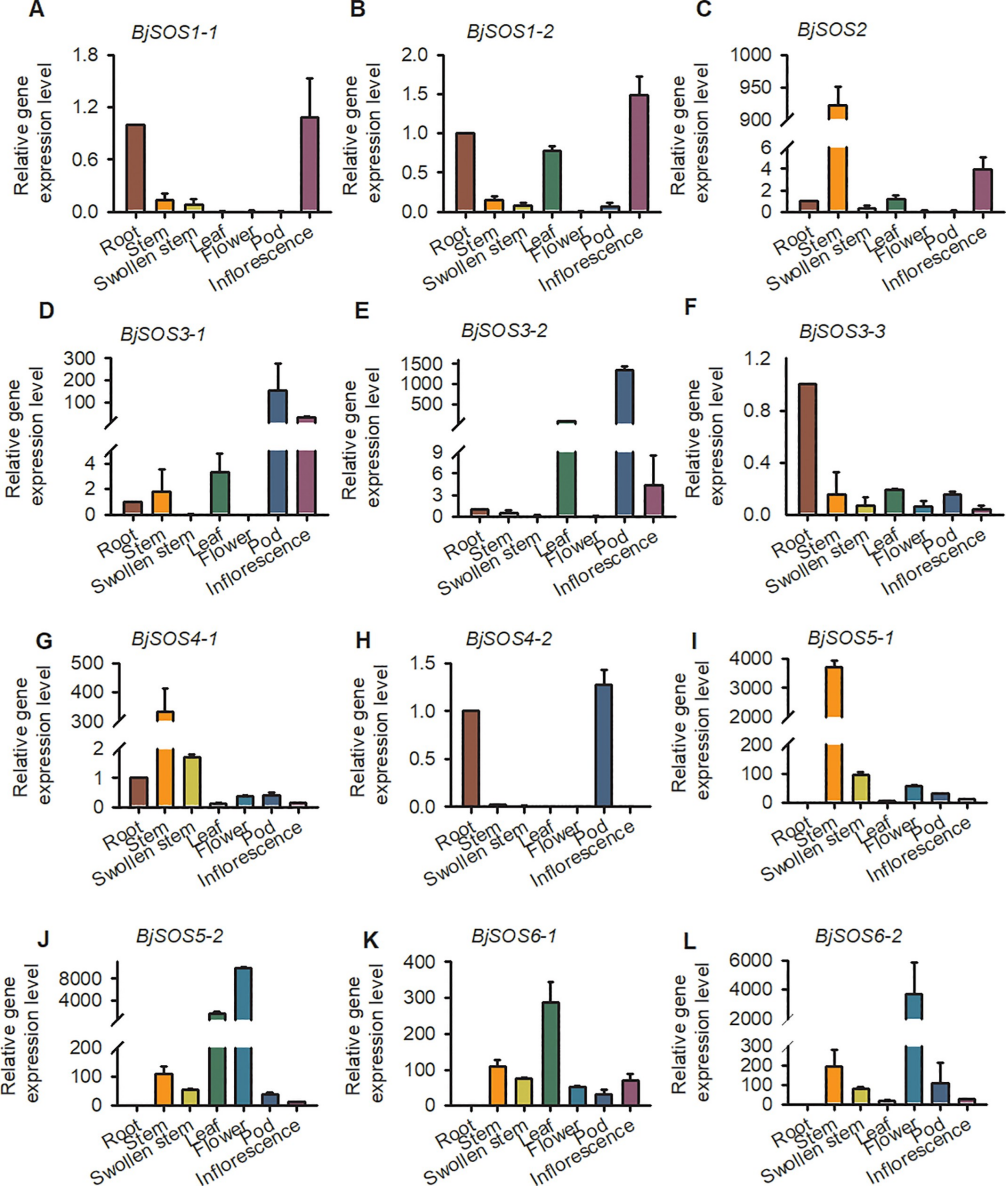
Expression levels of SOS homologs genes in different tissues. Tissue specific expression pattern of SOS homologs genes were analyzed by qPCR. Data were normalized to the expression level of *BjActin3*. The values are means ± standard error. Three independent biological repeats were performed.

To investigate the tissue specific expression patterns of *SOS* homologs, we analyzed the gene expression levels at different growth stages and tissues (root, stem, swollen stem, leaf, pod and inflorescence) using qRT-PCR. The results showed that the *SOS1* homolog *BjSOS1-2* was highly expressed in the root, leaf, and inflorescence. In contrast, the expression level of another homolog *BjSOS1-1* was very low, with nearly no expression in pod; *BjSOS2* was highly expressed in the stem; the *SOS3* homologs *BjSOS3-1* and *BjSOS3-2* were highly expressed in the leaf, pod, and inflorescence, whereas there was very low *BjSOS3-3* expression in *B*. *juncea* var. *tumida* (Fig 6). *BjSOS4-1* and *BjSOS5-1* was highly expressed in stem; *BjSOS5-2* was highly expressed in leaf and flower; and the expression level of *BjSOS6-1* and *BjSOS6-2* were high in almost all the tissues (Fig 6). According to the results, the expression levels of the *SOS* homologs varied in different tissues and organs, indicating that they may play different roles in different tissues. In addition, the different expression patterns of the same gene in different tissues and organs suggest that the expression pattern of the genes was existence of space-time specificity.

### Gene expression levels of SOS homologs in *B*. *juncea* var. *tumida* under abiotic and biotic stresses

**Fig 7:**
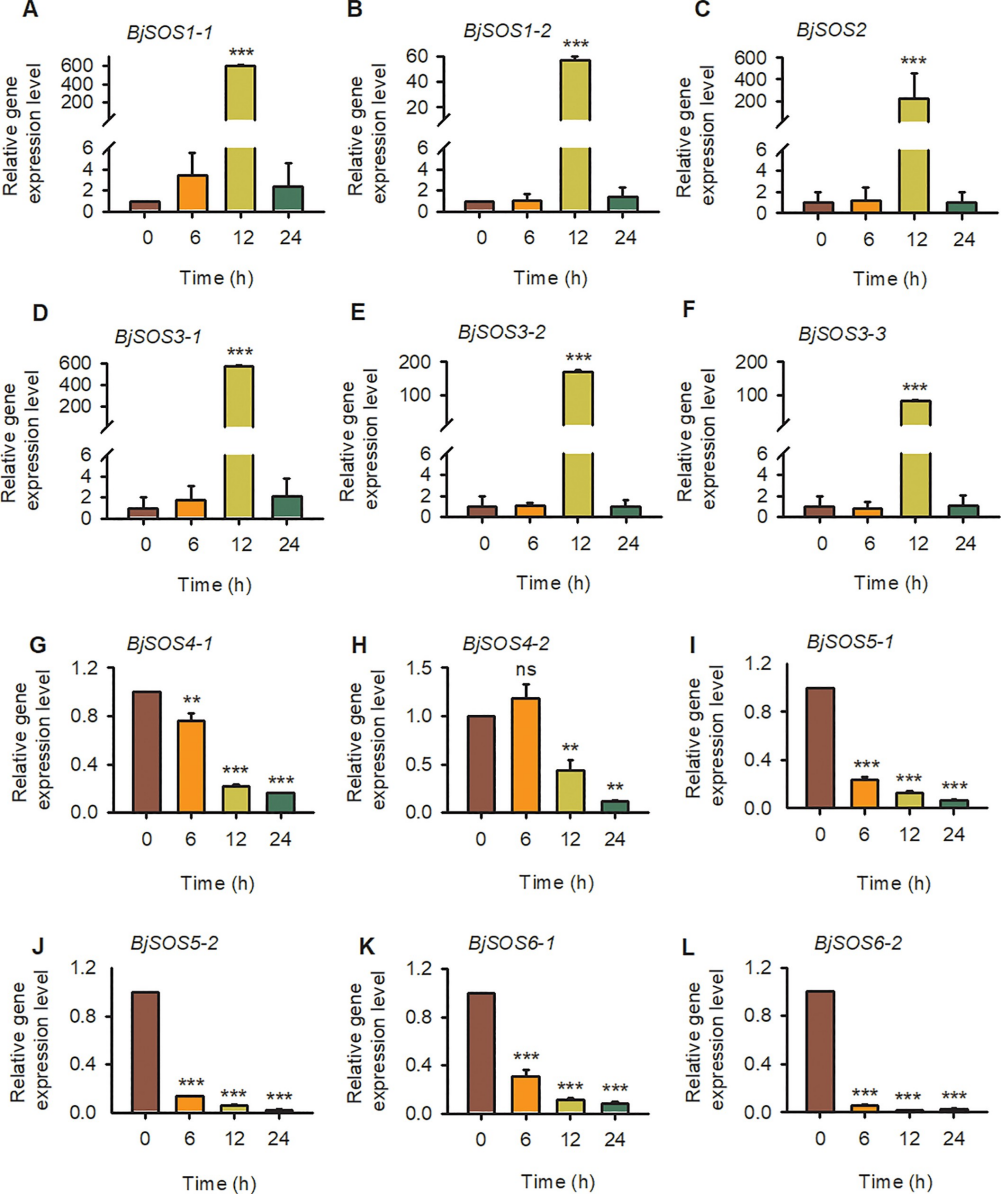
The expression patterns of SOS homologs genes under salt stress. Total RNA was extracted from tuber mustard seedlings treated with 200 mM NaCl at the indicated time points. Data were normalized to the expression level of *BjActin3*. The values are means ± standard error. Three independent biological repeats were performed.

**Fig 8:**
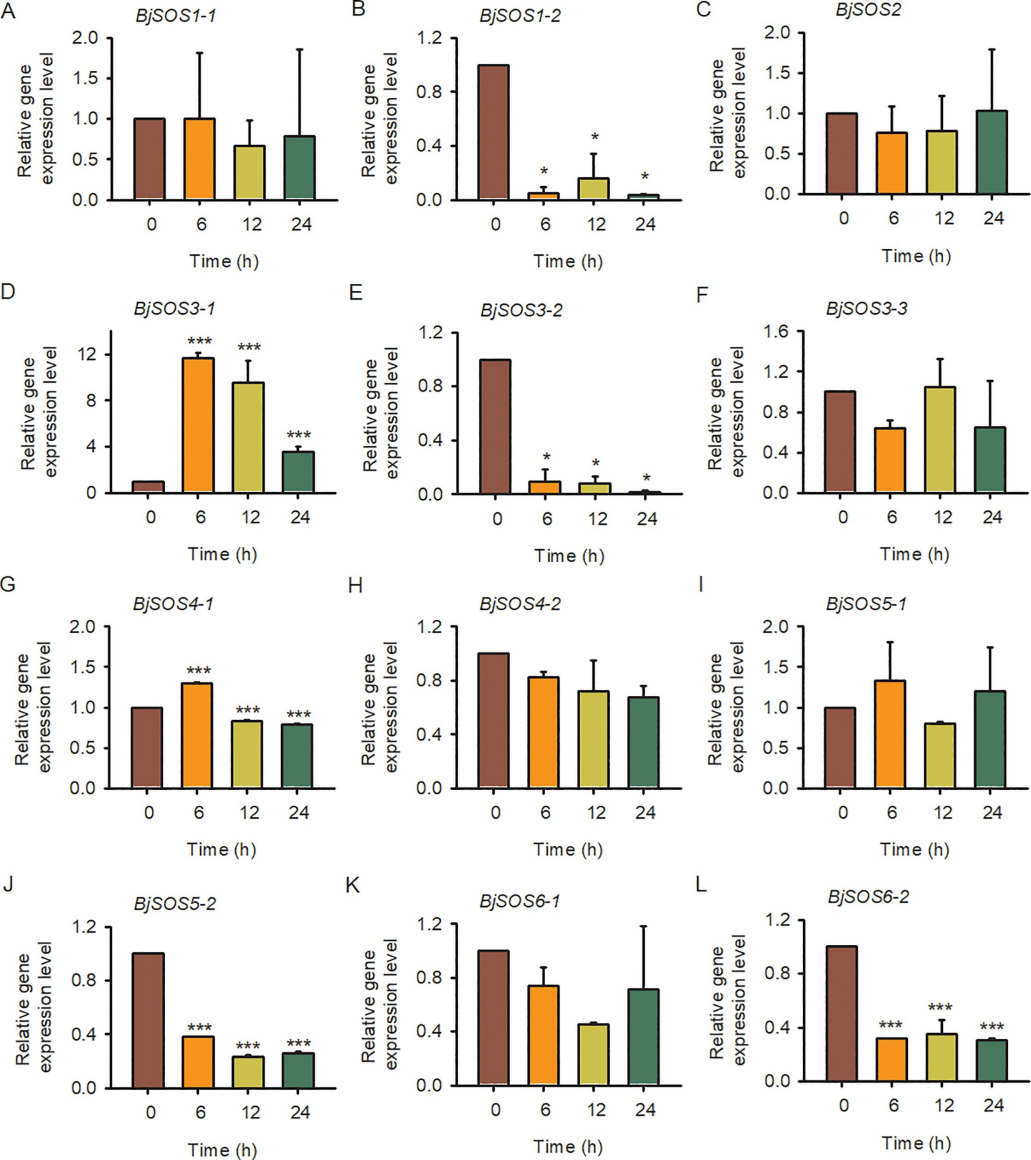
The expression patterns of SOS homologs genes under ABA treatment. Total RNA was extracted from tuber mustard seedlings treated with 50 μM ABA or not at the indicated time points. Data were normalized to the expression level of *BjActin3*. The values are means ± standard error. Three independent biological repeats were performed.

**Fig 9:**
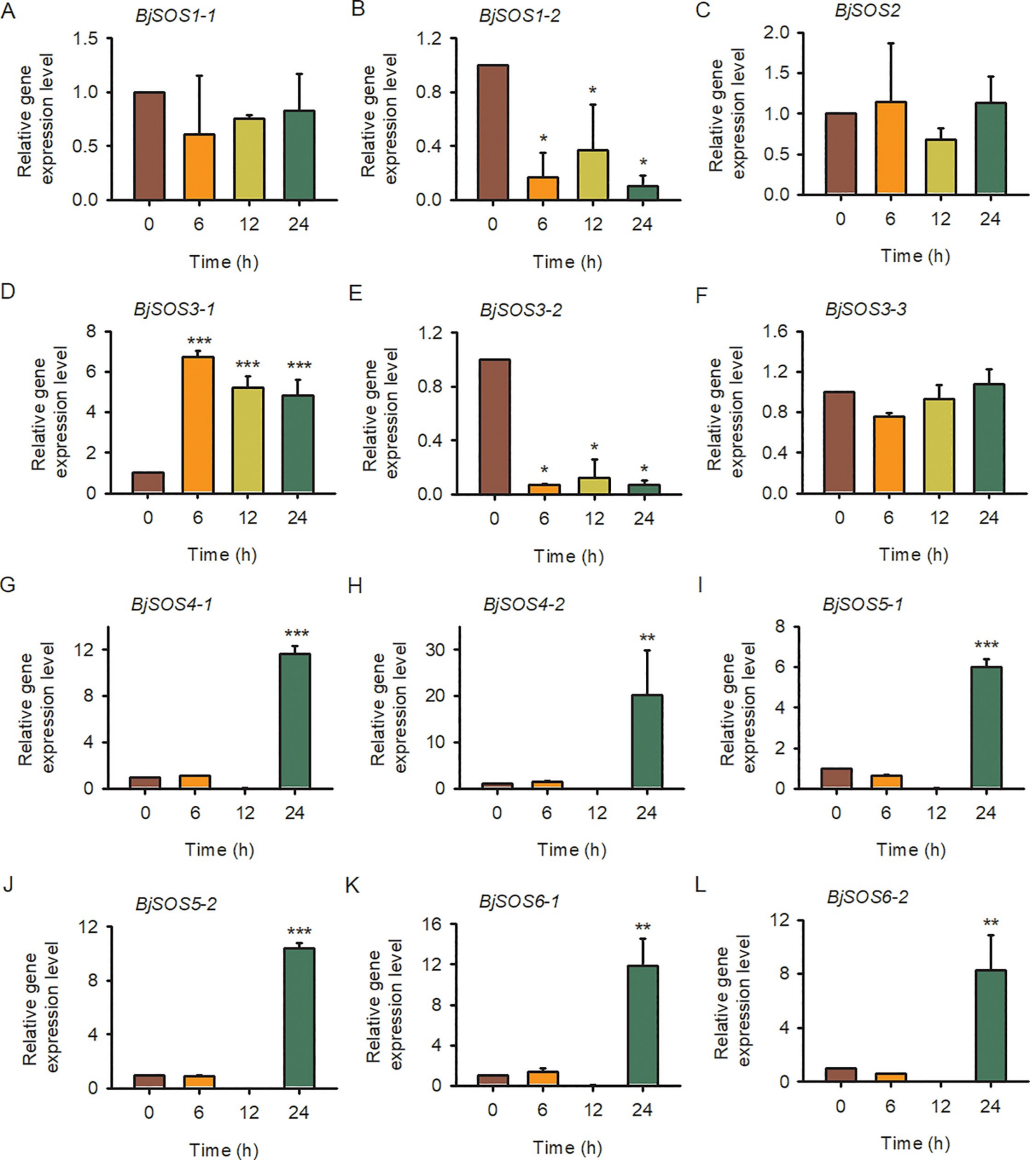
The expression patterns of SOS homologs genes under low tempreture treatment. Total RNA was extracted from tuber mustard seedlings treated with 4°C or not at the indicated time points. Data were normalized to the expression level of *BjActin3*. The values are means ± standard error. Three independent biological repeats were performed.

**Fig 10:**
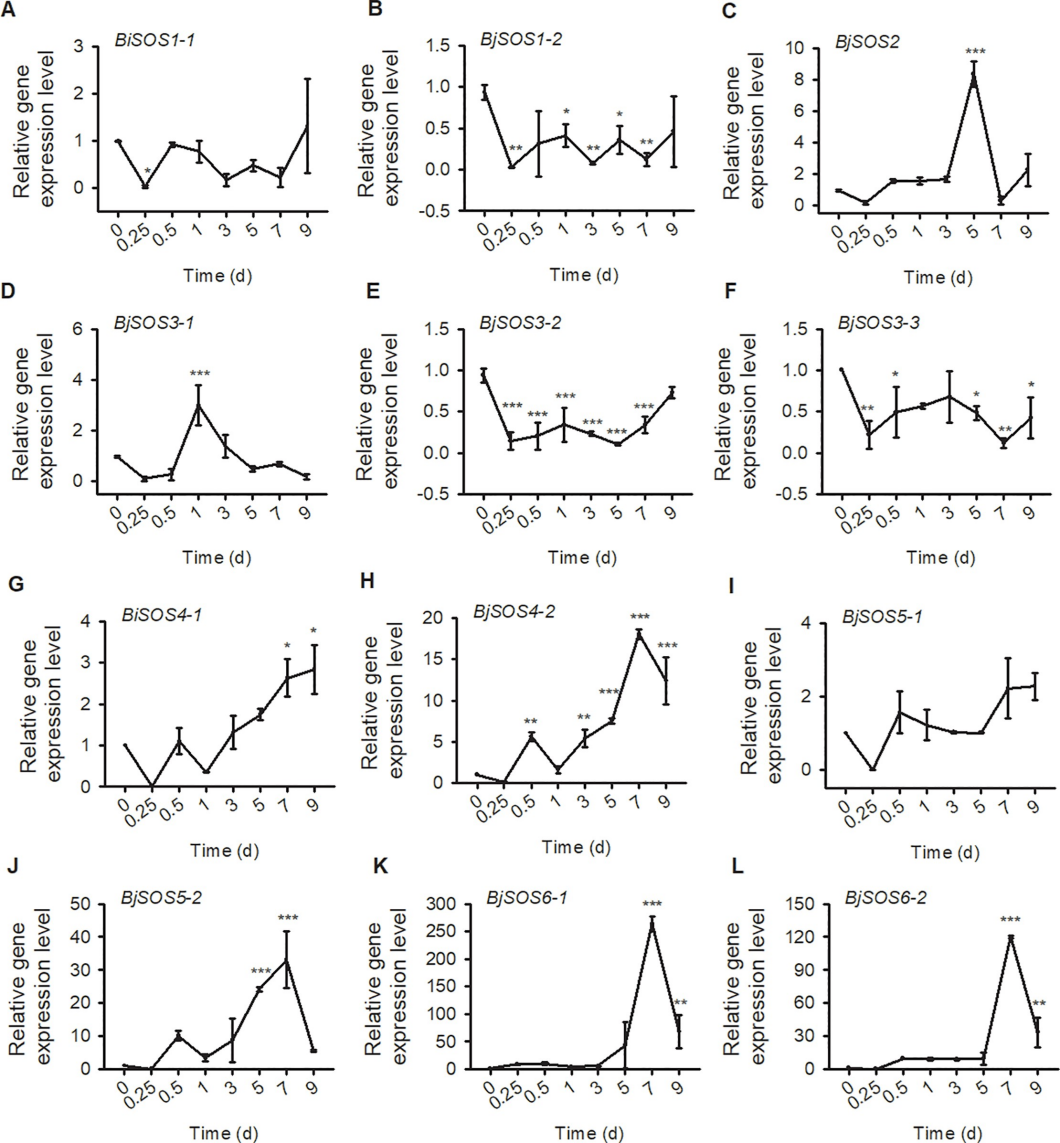
The expression patterns of SOS homologs genes under pathogen treatment. Total RNA was extracted from tuber mustard seedlings treated with *Plasmodiophora Brassicae* at the indicated time points. Data were normalized to the expression level of *BjActin3*. The values are means ± standard error. Three independent biological repeats were performed.

To further explore the *SOS* homolog expression levels in tuber mustard under biotic and abiotic stresses, qRT-PCR was performed using tuber mustard seedlings treated with 200 mM NaCl, 50 μM ABA, low temperature (4°C) and the pathogen *P*. *brassicae* at different time points. Under NaCl treatment, all of the *BjSOS1-1*, *BjSOS1-2*, *BjSOS2*, *BjSOS3-1*, *BjSOS3-2* and *BjSOS3-3* genes were induced by salt stress, especially at 12 h after NaCl treatment, and then decreased to normal level at 24 h; and all of the *BjSOS4-1*, *BjSOS4-2*, *BjSOS5-1*, *BjSOS5-2*, *BjSOS6-1* and *BjSOS6-2* genes were repressed by salt stress, indicating that these *SOS* homologs play important roles in the plant response to salt stress (Fig 7). The expression levels of *BjSOS3-1* and *BjSOS4-1* were significantly induced after ABA treatment. In contrast, the gene expression level of *BjSOS1-2*, *BjSOS3-2*, *BjSOS5-2* and *BjSOS6-2* were downregulated under ABA treatment, suggesting that *BjSOS1-2*, *BjSOS3-1*, *BjSOS3-2*, *BjSOS4-1*, *BjSOS5-2* and *BjSOS6-2* might be involved in the ABA signaling pathway (Fig 8). Under low temperature stress condition, the gene expression level of *BjSOS3-1*, *BjSOS4-1*, *BjSOS4-2*, *BjSOS5-1*, *BjSOS5-2*, *BjSOS6-1* and *BjSOS6-2* were significantly induced and the transcript levels of *BjSOS1-2* and *BjSOS3-2* were downregulated. However, there was no obvious expression difference at 0, 6, 12 and 24 h after 4°C treatment of *BjSOS1-1*, *BjSOS2*, and *BjSOS3-3*, indicating that *BjSOS1-2*, *BjSOS3-1*, *BjSOS3-2*, *BjSOS4-1*, *BjSOS4-2*, *BjSOS5-1*, *BjSOS5-2*, *BjSOS6-1* and *BjSOS6-2* regulated the tuber mustard response to low temperature stress (Fig 9). Under pathogen stress, we treated the tuber mustard seedlings with *P*. *brassicae* for 0, 0.25, 0.5, 1, 3, 5, 7, and 9 days, and the qRT-PCR results showed that *BjSOS3-1* was induced by pathogen on day 1; the *SOS2* homolog *BjSOS2* was upregulated by pathogen, especially on day 5, and then downregulated at later time points; *BjSOS4-2*, *BjSOS5-2*, *BjSOS6-1* and *BjSOS6-2* were up-regulated by pathogen, especially on day 7, suggesting that *BjSOS3-1*, *BjSOS2*, *BjSOS4-2*, *BjSOS5-2*, *BjSOS6-1* and *BjSOS6-2* may play crucial roles in biotic tolerance (Fig 10). Taken together, the expression patterns of *SOS* homologous genes changed under salt, ABA, 4°C, and pathogen treatment, indicating that these genes in tuber mustard might be important candidates for regulating plant tolerance to biotic and abiotic stresses.

## Discussion

The SOS signaling pathway plays important roles in the plant response to salt stress; however, the identity and expression patterns of *B*. *juncea* var. *tumida SOS* genes are unknown. Here, a total of twelve *BjSOS* genes were identified and located in 9 of 18 chromosomes (Fig 1). There was high sequence identity between BjSOSs and their relative homologs in *A*. *thaliana*, and they shared the same protein motifs (Fig 4). The expression patterns of *BjSOS* genes indicated that they might play specific roles in different tissues and stress conditions. These results shed light on the roles of *BjSOS* genes in regulating plant growth and response to abiotic and biotic stresses, which will be helpful for improving the production of tuber mustard.

In *A*. *thaliana*, six *SOS* genes *SOS1*, *SOS2*, *SOS3*, *SOS4*, *SOS5* and *SOS6* were identified [2–5, 7–10]. According to our results, twelve *SOS* homologs were found in the genome of *B*. *juncea* var. *tumida*. There were more members of the SOS genes in the *B*. *juncea* var. *tumida* genome than in *A*. *thaliana*, most likely because *B*. *juncea* var. *tumida* is an allotetraploid species that resulted from the hybridization between *B*. *rapa* and *B*. *nigra* following with genome duplication [1]. The comparable homologous gene number in the A sub-genome and B sub-genome indicated the *B*. *juncea* var. *tumida* genome experienced co-linearity gene duplication. However, the homologous genes of *AtSOS2* were lost or not duplicated in *B*. *juncea* var. *tumida*, suggesting that these homologous genes may have had functional redundancy or divarication during the evolutionary process. The loss of genes during the genome duplication event has also frequently occurred in other *Brassica* species, such as chitinase gene family in *B*. *rapa* [25].

The *cis*-elements and functional characterization of the promoters of *SOS* genes have been identified in many species such as *Salicornia brachiate*, *B*. *juncea*, and *A*. *thaliana* [26–28]. In this study, we analyzed the promoter *cis*-elements of *SOS* homologs in *B*. *juncea* var. *tumida*, and found that all of the promoters contained diverse *cis*-elements responsive to plant hormones (ABA, auxin, GA, and SA) and abiotic stresses (drought, cold, and salt stresses) (Fig 5). These promoter *cis*-elements of *SOS* genes in *B*. *juncea* var. *tumida* are in accordance with previous studies in other species [26–28], indicating that the expression patterns of *SOS* homologs were regulated by hormone and abiotic stresses, and that these homologs might play roles in regulating the tuber mustard response to hormone and abiotic stresses.

To date, although the expression patterns of *SOS* family genes have been determined in other species, such as wheat, *Arabidopsis* and Brassica [12, 14, 27–30], no detailed studies on the expression of *BjSOS* genes were found. Here, tissue specific expression pattern analysis results revealed that majority of *BjSOS* genes expressed in various tissues, such as root, stem, pod, leaf and flower (Fig 6). Notably, some genes highly expressed in root, stem and leaf, pointing to the important roles of *BjSOS* genes in those tissues and the diverse biological functions of different *BjSOS* genes in *B*. *juncea* va**r**. *tumida*. In *Arabidopsis*, *AtSOS1* promoter-driven GUS expression was mainly found in root, inflorescence and leaf [31], similar tissue specific expression patterns were also found for *BjSOS1* genes, suggesting that *SOS1* genes played conserved functions in *Arabisopsis* and tuber mustard (Fig 6). In *Arabidopsis*, *AtSOS4* expressed ubiquitously in all organs, in contrast, *BjSOS4-1* mainly expressed in stem, while almost no expression of *BjSOS4-2* could be detected in stem, leaf and flower (Fig 6), indicating the different roles of *BjSOS4* genes in the regulation of tuber mustard growth and development [7, 8]. The expression patterns of *BjSOS* genes under different stress treatments were examined by qPCR and found that they were differentially expressed after different stress treatments. According to the results of gene expression pattern analysis, all of the *SOS* homologs were significantly response to salt stress; *BjSOS3-1* was induced by ABA and low temperature; *BjSOS4-1*, *BjSOS4-2*, *BjSOS5-1*, *BjSOS5-2*, *BjSOS6-1* and *BjSOS6-2* were induced by low temperature, *BjSOS2*, *BjSOS3-1*, *BjSOS4-2*, *BjSOS5-2*, *BjSOS6-1* and *BjSOS6-2* were significantly induced by treatment with the pathogen *P*. *brassicae*, indicating that *BjSOS* genes might regulate the tuber mustard response to abiotic and biotic stresses (Figs 7–10). In rice, *OsSOS1* was highly induced in root after 15 h salt stress treatment comparing with non-treated plant [32], in accordance with this, both the expression levels of *BjSOS1-1* and *BjSOS1-2* were significantly induced in root by salt treatment at 12 h (Fig 7), and the expression induction was also found for *PabSOS1 Populus* [33]. Besides that, *AtSOS1* was significantly induced by NaCl but not ABA and cold [2], similar expression patterns were also found for *BjSOS1-1* and *BjSOS1-2* (Figs 7–9), those results suggested that the functions of *SOS1* genes are conserved in the regulation of plant response to salt stress in different species. Although abiotic and biotic stresses responsive *cis*-elements could be found in the promoters of some *BjSOS* genes, the expressions of those genes were not induced by ABA, low temperature or pathogen. The event of gene expression level not in agreement with the promoter analysis of *cis*-element also frequently exists in other species, such as *BnPYLs* in *B*. *napus* and *BjuTIR1/AFBs* in *B*. *juncea* var. *tumida* [34–35].

In conclusion, our study identified twelve *BjSOS* genes in tuber mustard and analyzed their transcript levels under the biotic and abiotic stresses. The results suggest that SOS family genes might potentially be utilized for improving the tolerance of *B*. *juncea* var. *tumida* to biotic and abiotic stresses.

## Supporting information

S1 Table The primers used in this study. (DOCX)

S2 Table Number of elements responsive to stresses and hormones in the promoter regions of *BjSOS* genes. (DOCX)
