# Supplementary material for: Genome-wide identification and gene expression analysis of SOS family genes in tuber mustard (Brassica juncea var. tumida)
Source: PLoS One. 2019 Nov 11;14(11):e0224672. doi: 10.1371/journal.pone.0224672 (PMC6844470; doi:10.1371/journal.pone.0224672)
Supplement: S1 Table — (DOCX) [file pone.0224672.s001.docx]

S1 Table The primers used in this study

| name | Homologous gene | primer |
| --- | --- | --- |
| *AtSOS1* | *BjSOS1-1* | F:GCTGCGTTTGCAAGGACAGC |
|  |  | R:GAAAAAGAAATCCCCATGAATTC |
|  | *BjSOS1-2* | F:GTAATAAGGCAGATGTTCCT |
|  |  | R:ACTGTTCACGGTAATAGCATAAG |
| *AtSOS2* | *BjSOS2* | F:CAAGGCTGGAGGGTTTATCTT |
|  |  | R:TCACCAGCAGCCTTTCTAAC |
| *AtSOS3* | *BjSOS3-2* | F:CAGAAATAGGAACCAGAAG |
|  |  | R:CAGTTTGCCTTAAGTCATAC |
|  | *BjSOS3-1* | F:GTTATTCAGAAATAGAAACCG |
|  |  | R:CGCCTAGAGACCTGACAAACTCC |
|  | *BjSOS3-3* | F:CTCGGAAACAGGAACCGGAAC |
|  |  | R:ATGGAAGACACCTAAAGACCGAAC |
| *AtSOS4* | *BjSOS4-1* | F: GCTCTTCTACTTGGTTGGAGTAA |
|  |  | R: TACCCGGCTCGTTTGTAATC |
|  | *BjSOS4-2* | F: GGCTTCTATGCTGACTCCTAAC |
|  |  | R: GGACCAGCTGCATGAAGAATA |
| *AtSOS5* | *BjSOS5-1* | F: CTTCCTTCTCCAACCTCCTTG |
|  |  | R: GCGGAGGAGAAGTGAGAATTAG |
|  | *BjSOS5-2* | F: CCATCTCGCATCCGTCTATTT |
|  |  | R: AGGAGGTTGGAGAAGGAAGA |
| *AtSOS6* | *BjSOS6-1* | F: TAGGAGGAGGTCTGATGCTTAT |
|  |  | R: GTGGCCTTCGCAACTTTAAC |
|  | *BjSOS6-2* | F: GCCGTTCTTCAAGGTCTTCT |
|  |  | R: CAAACTCGTCGTCTCCATCTT |
| Reference gene | *BjActin3* | F:GGCTACTCTTTCACCACGAC |
|  |  | R:GGATACCAGCATTCTCCATAC |
