# Supplementary material for: Genome-wide identification and gene expression analysis of SOS family genes in tuber mustard (Brassica juncea var. tumida)
Source: PLoS One. 2019 Nov 11;14(11):e0224672. doi: 10.1371/journal.pone.0224672 (PMC6844470; doi:10.1371/journal.pone.0224672)
Supplement: S2 Table — (DOCX) [file pone.0224672.s002.docx]

S2 Table Number of elements responsive to stresses and hormones in the promoter regions of *BjSOS* genes

| Elements  Genes | Hormone-relative elements | | | | | | | | Stress-relative elements | | | | | | |
| --- | --- | --- | --- | --- | --- | --- | --- | --- | --- | --- | --- | --- | --- | --- | --- |
|  | Gibberellin | | | Abscisic acid | MeJA | | Auxin | Salicylic acid | Drought | | | | Pathogen and salt | Defense and stress | Low temperature |
|  | TATC-box | p-box | GARE-motif | ABRE | CGTCA-motif | TGACG-motif | TGA-element | TCA-element | MYC | DRE | MBS | MYB-like sequence | GT1GMSCAM4 | TC-rich repeats | LTR |
| *BjSOS1-1* |  | 1 |  | 2 |  |  | 1 |  | 3 | 1 | 1 |  | 7 |  |  |
| *BjSOS1-2* | 1 |  |  |  |  |  |  |  | 3 |  | 1 | 5 | 4 |  |  |
| *BjSOS2* |  |  |  |  |  |  |  |  | 3 |  | 1 | 1 | 7 | 1 | 1 |
| *BjSOS3-1* |  | 1 |  | 2 | 3 | 2 | 1 | 2 |  |  |  | 4 | 4 |  | 1 |
| *BjSOS3-2* |  |  |  | 10 | 2 | 2 | 1 |  | 4 |  | 1 | 6 | 5 |  |  |
| *BjSOS3-3* |  |  | 1 | 2 | 2 | 2 | 1 |  | 1 |  |  | 8 | 2 |  |  |
| *BjSOS4-1* | 1 |  |  |  | 1 | 1 |  | 1 | 5 |  | 1 | 3 | 7 |  |  |
| *BjSOS4-2* |  |  |  | 1 | 2 | 2 |  |  | 4 |  |  | 1 | 8 | 1 | 1 |
| *BjSOS5-1* |  |  |  | 1 |  |  |  |  | 5 |  | 1 | 2 | 2 | 1 | 1 |
| *BjSOS5-2* |  |  |  | 2 |  |  |  | 1 | 6 | 1 |  |  | 1 |  |  |
| *BjSOS6-1* |  | 1 | 1 |  | 2 | 2 |  | 2 | 4 |  |  | 3 | 2 | 1 |  |
| *BjSOS6-2* |  | 1 |  | 2 | 1 | 1 |  | 2 | 4 |  | 2 | 5 | 3 |  |  |
